# The Roles of Personal and Environmental Resources in Predicting Work–Family Facilitation and Mental Health among Employed Parents of Children with Disabilities in Croatia

**DOI:** 10.3390/bs13090710

**Published:** 2023-08-27

**Authors:** Ana Šimunić, Ana Slišković, Andrea Tokić, Jelena Ombla, Lisa Stewart

**Affiliations:** 1 Department of Psychology, University of Zadar, 23000 Zadar, Croatia; aslavic@unizd.hr (A.S.); apupic@unizd.hr (A.T.); jlevac@unizd.hr (J.O.); 2 Department of Social Work, California State University, Monterey Bay, Long Beach, CA 93955-8001, USA; listewart@csumb.edu

**Keywords:** parents of children with disabilities, mental health, work–family facilitation, emotional regulation strategies, optimism, posttraumatic growth, social support, recovery management

## Abstract

Quantitative research on the positive aspects of work–life integration and the well-being of families with children with disabilities is scarce, especially in the national context. The family can provide gains that enhance work domain functioning (family-to-work facilitation; FWF), and work can provide gains that enhance family domain functioning (work-to-family facilitation; WFF). The aim of this study is to examine the contributions of some personal and environmental resources in explaining WFF and FWF and the mental health of parents of children with disabilities residing in Croatia. The mediational role of WFF and FWF in the relationship between resources and mental health was tested, while controlling for some general socio-demographic variables. A total of 571 employed parents of a child/children with disabilities completed an online self-assessment questionnaire. The results show that higher WFF (19%) was predicted by higher levels of social support at work, a higher level of education, posttraumatic growth (PTG) of personal strength, and recovery management. Higher FWF (46%) was predicted by higher levels of social support in the family, PTG of personal strength, the emotional regulation strategy of reorienting to planning, optimism, a younger age, the male gender, a greater number of children, and a higher level of education. A higher level of mental health (47%) was predicted directly by higher levels of optimism, recovery management, FWF, emotional regulation strategies of positive refocusing and planning, and a greater number of children, and was indirectly predicted by all the predictors of FWF through a higher level of FWF (but not WFF).

## 1. Introduction

Mental health implies “a state of mental well-being that enables people to cope with the stresses of life, realize their abilities, learn well and work well, and contribute to their community” [1]. The birth of a child with a developmental risk and/or learning that one’s child has a developmental disability is a traumatic event for parents that requires resources to overcome the trauma and cope with the numerous challenges of caring for a child with an atypical developmental trajectory [2,3]. The challenges include learning about a specific difficulty; finding access to effective treatments; coordinating the implementation of various diagnostic, therapeutic, and intervention procedures; advocating the child’s rights in social, health, and educational contexts; paying for interventional and therapeutic procedures that are not covered by health insurance; and dealing with the high physical and emotional demands of care. When it comes to parents simultaneously being employed, research results suggest that balancing family and work roles is more difficult for parents of children with disabilities than for parents of typically developing children, and that their employment may be associated with lower levels of well-being and health [4,5]. However, some research results suggest that employment promotes parental well-being since it could serve as a distraction from their complex family environment [6,7]. The issues of integrating work and family roles have been extensively studied in the general population, mostly through the negative aspects in terms of the experiences of work–family conflict and imbalance [8,9,10]. Brown and Clark reviewed 54 papers dealing with the work and family role balance in parents of children with disabilities up to 18 years of age and warned about the lack of serious quantitative research and research on the positive aspects of work–family integration, that is, how being employed facilitates family life and vice versa [6]. Additionally, the papers in this review come from countries with heterogeneous organizational policies aimed at balancing work and family roles, so national research on this topic is necessary. This was also pointed out in the review by Slišković et al. [11]. The application of the principles of positive psychology to the field of developmental disabilities suggests the need to focus on the strengths, possibilities, and opportunities of children with developmental disabilities and their families, instead of the prevailing perspective that is focused on deficits, stressors, and problems [12,13]. Therefore, in this study, we decided to focus on the positive aspects and explore some personal and environmental strengths and resources that could promote the experiences of work–family facilitation and mental health in parents of children with disabilities in the Croatian national context.

Parents of children with disabilities, although exhausted by the demands of care and struggling with the many obstacles they encounter in everyday life, report many positive aspects of parenting children with disabilities [14,15,16,17,18,19]. Seeing a satisfied and happy child who has made even the smallest progress in development, which is normally “taken for granted”, is what gives parents strength and contributes to their personal growth and well-being. The positive changes detected in parents of children with developmental disabilities could be paralleled to those found in the model of posttraumatic growth (PTG) [20], reflecting three broad domains: changes in self-perception, interpersonal relationships, and a changed philosophy of life. PTG reflects the growth that occurs after someone struggles with challenging events that are theorized to disrupt the fundamental assumptions about the world being safe, benevolent, and predictable [21]. In line with the PTG model, past research indicates that certain events (e.g., disability diagnosis) may profoundly shock an individual [22] and act as catalysts for initiating the growth process (e.g., for parents of children diagnosed with Down syndrome) [23]. In a one-year qualitative study of parental distress and personal growth [24], parents expressed distress and grief while using coping strategies and resources to manage demands and optimize opportunities for growth. Emotional, cognitive, and behavioral adaptations to the child’s condition proved to be important factors for the well-being of parents of children with disabilities [25].

Another individual protective factor in parents of children with disabilities was the use of adaptive stress coping strategies [17,26] and/or effective emotional regulation strategies [27]. Emotional regulation is an important factor that affects a person’s well-being and successful functioning, as well as one’s mental health. The construct of emotional regulation is close to the construct of coping, but emotional regulation refers to both the regulation of negative and positive emotions, while coping is somewhat narrower and refers to a person’s ability to deal with stressful situations and reduce negative emotions [28]. There should also be a differentiation between cognitive and behavioral strategies since cognitive processes precede action. Cognitive emotional regulation strategies such as positive reappraisal, putting things into perspective, positive refocusing, and planning were shown to help people more easily tolerate and overcome negative life experiences [29]. Among mothers of adolescents with developmental disabilities, it was shown that coping skills such as active coping/planning, positive reinterpretation/growth, and behavioral/mental disengagement minimized the effects of stressful parent–child interactions [30].

Behavioral and mental disengagement can be viewed through the broader concept of recovery. Recovery refers to the restoration processes of an individual’s increased strain level as a reaction to a stressor or demand, returning to the previous level [31]; managing to recover from this increased strain level has been associated with greater well-being [32]. Geurts and Sonnentag [33] addressed the recovery phenomena and presented the Effort–Recovery Model and the Allostatic Load Theory [34] as unifying frameworks. Sonnentag and Fritz argued that specific experiences that help with mood repair should underlie effective recovery processes [35]. In this context, researchers look at activities (i.e., what people do during their non-work time) and experiences (i.e., what psychological state people are in during their non-work time). When family demands are very high, opportunities for recovery might be very limited, and yet very important [36].

In a qualitative study, semi-structured interviews were conducted on 25 employed parents of children with developmental disabilities living in Croatia [37]; the participants were to discuss the factors they believe promote their well-being and help them balance work and private roles. Most of them said that even the slightest departure from everyday life helps them, such as going out for coffee alone or with friends, while some parents relax through sports and recreation. Of the personality characteristics that help them balance family and work obligations daily, the parents reflected on optimism, emotional stability, patience, combativeness, and organization. Optimism was noticeably shown to predict a higher overall well-being in other studies on parents of children with developmental disabilities as well. Higher levels of optimism predicted increased positive feelings and decreased negative feelings, even after controlling for the levels of parenting stress and child problematic behavior [38]. In parents of autistic children, higher levels of optimism predicted lower levels of depressive symptoms, but was only protective for mothers (not for fathers) [39]. In the aforementioned study [37], the parents also generally pointed out the benefits of different forms of social support (emotional and instrumental) from various sources (family members, friends, workplace supervisors and colleagues, and the wider society). Social support was also found to be a protective factor of the well-being of this group of parents in other studies, especially in the family [26,40], and was related to work–family balance [6].

Much of the research on work–family/life integration has focused on theoretical models that include individual, work, and family characteristics as antecedents (e.g., structural and psychological stressors, demands, resources, and support), mediator variables related to the interface (positive and negative) of work and family roles, and outcome variables related to the well-being of the individual, family, and organization [41,42]. In its essence, work–family facilitation is the sense that work and family are complementary, that is, the involvement in one domain can beneficially influence the functioning of the other domain. It can occur bidirectionally; family can provide gains that enhance work domain functioning (family-to-work facilitation or FWF), and work can provide gains that enhance family domain functioning (work-to-family facilitation or WFF) [43]. Using the Family Resilience Theory, Grzywacz and Bass studied the effects of work–family facilitation on the mental health of working adults and showed that adult mental health is optimized when work–family facilitation, especially FWF, is high [44]. This implies that factors promoting greater work–family facilitation could also promote a higher level of mental health. Wayne and colleagues (2007) offer the Resource–Gain–Development (RGD) perspective to explain facilitation and identify its primary antecedents, consequences, and moderators [45]. The RGD perspective specifies that personal characteristics and environmental resources that promote positive domain experiences and the acquisition of gains are the key promoters of facilitation. Such personal characteristics are the aspects of oneself that promote positivity and the tendency to experience positive emotional states, seek positive developmental experiences, and earn status and other assets. Environmental resources include object, condition, energy, and support resources that promote positive, dynamic, and enriching environments. Some individuals can obtain more resources from their environment or can more effectively use resources and thus receive greater benefits. Thus, theoretically speaking, all the above-mentioned promoters of well-being in parents of children with disabilities could be expected to also promote WFF and FWF. There is empirical evidence that some factors directly or indirectly promote well-being, although not specifically for parents of children with disabilities, except for social support and work–family balance [6]. In research on frontline hotel employees [46], work social support enhanced both directions of work–family facilitation, and family social support increased FWF. Both directions of facilitation were associated with higher work performance in the workplace. In research on police officers [47], optimism fostered work-to-family and family-to-work enrichment and psychological health. Work–life balance was shown to be impaired by the lack of recovery activities [48]. In another study [49], the relationships between coping and facilitation varied depending on the source domain. Positive thinking was associated with higher WFF and FWF, direct action was associated with higher FWF, and advice seeking was related to higher WFF.

In a study on mothers of children with learning disorders, these mothers reported a higher level of family-to-work conflict and a higher level of WFF compared to mothers of children without learning disorders [50]. FWF predicted a greater level of a child’s secure attachment to their mother and a greater level of family cohesion. In another study [51], those with a greater work–family balance, that is, lower work–family conflict and higher work–family enrichment (facilitation), had a greater number of children and greater parental and job satisfaction, but lower satisfaction with observed social attitudes towards their disabled child in comparison to other groups of parents according to the levels of work–family conflict and facilitation.

The aim of this study is to examine the contributions of some individual and environmental resources in explaining work-to-family and family-to-work facilitation and the mental health of parents of children with disabilities residing in Croatia. We assumed that higher levels of optimism, posttraumatic growth (personal strength), cognitive emotional regulation strategies (positive reappraisal and reorienting to planning), recovery management, and work and family social support will predict higher levels of WFF, FWF, and mental health. We also examined whether WFF and FWF have mediating roles in explaining the relationship between the examined predictors and mental health. In examining these relationships, we controlled for some general socio-demographic variables: gender, age, education, and number of children. We assumed that there were correlations between all the predictors and between WFF and FWF.

## 2. Materials and Methods

### 2.1. Participants

The study sample consisted of 571 employed mothers (*n* = 517; 90.5%) and fathers (*n* = 54; 9.5%) who have children with developmental disabilities. A convenient sample was used, with all participants required to have a child with an objectively diagnosed developmental disability up to 19 years of age (the age of legal adulthood in Croatia is 18), be employed, and have residence in the Republic of Croatia. The participants were very heterogenous in terms of the type of employment, with 384 (67.8%) working full time, and 184 (32.2%) working part time. They were from various regions of the country, mostly from the city of Zagreb (*n* = 127; 22.2%) and Zagreb County (*n* = 38; 66.5%), Split County (*n* = 69; 12.1%), Zadar County (*n* = 55; 9.6%), Primorje-Gorski Kotar County (*n* = 42; 7.4%), Osijek-Baranja County (*n* = 38; 6.7%), and Istria County (*n* = 28; 4.9%), with 14 other regions being represented by 2 (Požega-Slavonia County) to 23 participants (Varaždin and Međimurje Counties). Some other socio-demographic characteristics are shown in Table 1.

### 2.2. Procedure

After obtaining approval from the Ethics Committee of the Department of Psychology of the University of Zadar, data were collected using an online questionnaire. The questionnaire was distributed through institutions working with children with disabilities and their parents such as the Croatian Welfare Institute, kindergartens, schools, hospitals, special hospitals, schools, and other rehabilitation institutions. Some participants were reached through social networks and various nationwide associations uniting parents of children with developmental disabilities. Participation in the research was voluntary and anonymous. Participants gave their consent to participate in the study by clicking next after being given the study description on the first page of the online form. They were also given a chance to send the research group their comments or questions through the email address provided or through the Facebook page created for the study.

The research was conducted as part of a project funded by the University of Zadar called “Well-being of employed parents of children with developmental disabilities” (IP.01.2021.16).

### 2.3. Measures

Mental health was assessed with the Croatian Brief Mental Health Inventory [52]. It comprised five questions aimed at measuring general mental health. Subjects were asked to rate the frequency of each described condition in the past month on a six-point scale (from 1—never to 6—constantly). The questionnaire included the following domains: general positive affect (“How often were you happy?”), anxiety (“How often were you very nervous?”), depression (“How often did you feel discouraged and sad?”/”How often did you feel calm and peaceful?”), and behavioral/emotional control (“How often did you feel so depressed that nothing could cheer you up?”). The total score is the average score of all items after the reversed coding of three items. Thus, a higher score indicates a higher level of general mental health. The Cronbach’s α in this study is 0.90.

Work-to-family and family-to-work facilitation was assessed with two subscales from the work–family spillover scale [53], translated and adapted by Buljan [54]. Each dimension was measured with four items. The work-to-family facilitation items assessed the extent to which the skills, behaviors, or positive moods from the work role have positively influenced one’s role in the family (e.g., “The skills you use on your job are useful for things you have to do at home”), while the family-to-work facilitation items assess the degree to which the positive moods, behaviors, sense of achievement, support, or resources provided at home positively influence a person’s work role (e.g., “The love and respect you get at home makes you feel confident about yourself at work”). Respondents assessed, on a 5-point scale (1—never; 5—always), how often they experience each described situation in a typical work week. The total score is the average score of each set of the four items, with a higher score indicating greater facilitation. In this study, the Cronbach’s alpha reliability coefficient for the work-to-family facilitation subscale is α = 0.82, and the Cronbach’s alpha for family-to-work facilitation is α = 0.76.

Optimism was assessed with a subscale from the Optimism–Pessimism Scale [55]; it is an adapted version of the Optimism and Pessimism Scale by Chang et al. [56]. The subscale consisted of 6 items (e.g., “I always look at things from the positive side”), and respondents assessed the extent to which each item applies to them on a 5-point scale (1—does not apply to me at all; 5—applies to me completely). The total score is the average of the scores of all items, with a higher result indicating greater optimism. The Cronbach’s α in this study is 0.86.

Emotional regulation strategies of positive refocusing and planning were assessed with two subscales from the Cognitive Emotion Regulation Questionnaire (CERQ) [57], translated and adapted by Soldo and Vulić-Prtorić [58]. The questionnaire assessed the cognitive strategies that a person uses after facing a negative, threatening, or stressful life event or situation. Each subscale consisted of four items. Positive refocusing refers to thinking about other, more pleasant things instead of the actual event (e.g., “I’m thinking about something nice instead of what happened”). Planning (reorienting to planning) refers to thinking about the steps that need to be taken for the individual to face the event (e.g., “I am planning what would be the best thing to do”). On a 5-point scale (1—never; 5—always), respondents indicated how often they use each of the described ways of thinking after an unpleasant experience. The score was calculated by averaging the corresponding scores for each of the cognitive strategies, with a higher score indicating a more frequent use of a particular strategy. In this study, the Cronbach’s alpha reliability coefficient for the positive refocusing subscale is α = 0.93, and the Cronbach’s alpha for the reorienting to planning subscale is α = 0.82.

Personal strength was assessed with the Personal Strength subscale from the Posttraumatic Growth Questionnaire [59], which is a translated and adapted version of the Posttraumatic Growth Inventory (PTGI) [60]. We adapted the instructions to the studied population, emphasizing that they assess the changes that occurred after being confronted with the child’s diagnosis. The subscale consisted of 4 items (e.g., “I discovered that I am stronger than I thought”). Respondents assessed the extent to which they have experienced the described change on a 6-point scale (0—I have experienced no change; 5—I have experienced change to an extremely high degree). The total score is the average of the assessments, with a higher score indicating a higher level of posttraumatic growth in terms of personal strength after the life event. The Cronbach’s α in this study is 0.89.

Social support at work and in the family was assessed using 8 items (4 for social support at work and 4 for social support in the family) that were originally part of the Social Support at Work and Family Scale [61]. We selected only the items reflecting the support of one’s private life in the work domain (e.g., “My co-workers (superiors, colleagues) understand my personal and family needs”) and of the work issues from family members (e.g., “I can speak of my work to my family members (spouse/partner, children, parents, etc.) without embarrassment”). The respondents assessed their level of agreement with each item on a 7-point scale (1—I do not agree at all; 7—I completely agree). The total score is the average score of the two sets of four items after reversely coding one item in each set, and a higher score indicates a higher level of perceived social support. The Cronbach’s α in this study is 0.83 for social support at work, and 0.84 for social support in the family.

Recovery management in terms of managing time for oneself and relaxation and enjoying free time was assessed using two items from the Recovery Management subscale of the Work–Life Crafting Scale (Peeters and Demerouti, 2014, according to Wepfer et al. [48]): “I make sure that I can relax during my time off (e.g., me-time, hobbies, sports)”, and “I make sure that I do things that I enjoy during my time off (e.g., social activities, sports)”. The third item of the original subscale, “I take care that the amount of work time and private time are balanced”, was excluded due to the conceptual overlap with work–life balance. Respondents were to think about the time they spent when they were not working and gave assessments on how often they perform what is described in a typical work week on a 5-point Likert scale (1—never; 5—always). The total score is the average of the two assessments, with a higher score indicating a greater level of managing to recover or enjoy time for oneself. The Cronbach’s α in this study is 0.92.

The socio-demographic part of the questionnaire included questions on gender, age, level of education, place of residence, marital status, number of children, number of children with disabilities, the level of the child’s disability, the type of profession, and the spouse’s/partner’s employment status.

### 2.4. Analytical Strategies

Preliminary statistical analyses such as reviewing descriptive statistics and bivariate correlations between variables were conducted using the program Statistica 14 [62]. Path analyses were then performed in the program Mplus 8.7 [63] to estimate the hypothesized model, suggesting personal characteristics (resources) such as optimism, emotional regulation strategies (planning and positive refocusing), and posttraumatic growth (personal strength); enjoying time for oneself; and environmental resources such as social support in the family and at work as indirect predictors of mental health through WFF and FWF. Relationships with covariates (age, gender, education, and number of children) were controlled. Correlations between independent variables and covariates were specified, as well as a correlation between WFF and FWF. The full mediation model was first estimated to avoid just identifying the model, and modification indices were followed to check if there should be additional paths from first-level independent variables and mental health (the main dependent variable). The maximum likelihood (ML) parameter estimation method, justified via the descriptive statistics, and the full information maximum likelihood (FIML) for missing data imputation (the default) were used. Model fit was examined using the χ2/df ratio, with values of up to 3 demonstrating good fit; the comparative fit index (CFI) and the Tucker–Lewis index (TLI), with values above 0.90 signifying good fit and values above 0.95 signifying very good fit; and the root mean square error of approximation (RMSEA) and standardized root mean residual (SRMR), with values of up to 0.08 suggesting good fit [64]. The model with satisfactory fit indices and no more modification indices were interpreted, and indirect relationships (mediations) were tested by using the bootstrap method (1000 simulations).

## 3. Results and Discussion

### 3.1. Preliminary Analyses

The descriptive statistics of the study variables were calculated and are shown in Table 2. All variable results’ distributions were normal according to the skewness and kurtosis values varying between -2 and +2 [65], except for gender, with values not surpassing the values of skewness up to 3 and kurtosis up to 8 suggested by Klin [66]. The obtained values were shifted towards higher results on the variables of gender (a much larger number of women than men), posttraumatic growth (personal strength), emotional regulation strategies of planning (slightly for positive refocusing), social support at work and in the family, and mental health. The results were slightly shifted towards lower results on the variables of age, number of children, and recovery management. There were from 0 to 7 missing data points per variable.

The bivariate Pearson correlations between the study variables were calculated and are shown in Table 3. The results show that all the main study variables (optimism; emotional regulation strategies of positive refocusing and planning; posttraumatic growth in terms of personal strength, recovery management, and social support at work and in the family; work-to-family facilitation and family-to-work facilitation; and mental health) have significant positive low to intermediate correlations, except for the correlation between the emotional regulation strategy of planning and recovery management. The lowest correlation is 0.11, which is between the emotional regulation (ER) strategy of planning on the one hand, and WFF and mental health on the other. The highest correlations are between social support in the family and FWF (0.56) and optimism and mental health (0.58). There are only a few significant correlations between the socio-demographic variables and the main variables. Men tend to report higher levels of recovery management, FWF, and mental health. Older participants tend to report higher levels of recovery management and lower levels of FWF. A higher level of education is associated with a lower level of personal strength and positive refocusing, and with a higher level of WFF. A greater number of children is associated with a higher level of optimism, emotional regulation strategies of positive refocusing and planning, personal strength, FWF, and mental health. The men are somewhat older than the women in the sample; an older age is associated with a higher level of education and a greater number of children, and a higher education is associated with a lower number of children. The values of the variance inflation factor (VIF), indicating collinearity in a multiple regression, with mental health as the dependent variable and the other variables as predictors, are from 0.06 (gender) to 0.10 (age) when it comes to the socio-demographic variables. The lowest VIF for the main variables is 0.16 (emotional regulation strategy of planning), and then from 0.24 (work-to-family facilitation) to 0.32 (social support at work); the values in between are for positive refocusing and recovery management. The highest VIF values are 0.44 for optimism, 0.46 for social support in the family, and 0.47 for FWF. When looking at the VIF values without WFF and FWF as predictors, the values range from 0.05 (gender) to 0.07 (number of children) for the covariates, and from 0.13 (reorienting to planning) to 0.32 for social support in the family and 0.41 for optimism.

### 3.2. Testing the Hypothesized Model

**Figure 1:**
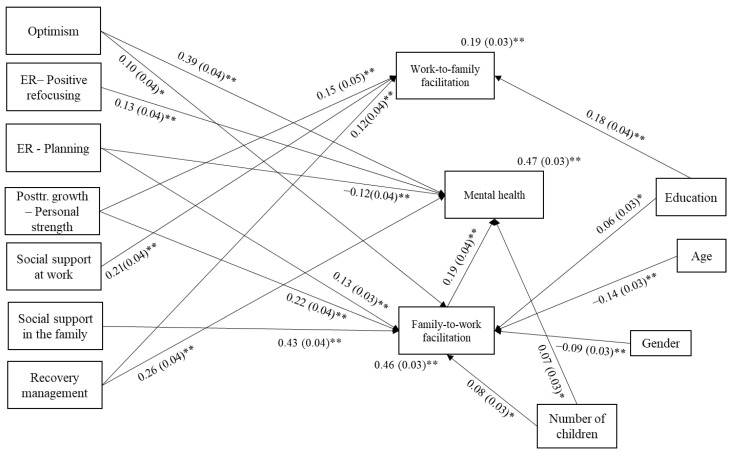
Display of the significant standardized (STDYX) path coefficients and standard errors (in brackets) of the tested relationships between the model variables (* *p* < 0.05; ** *p* < 0.01; *N* = 571).

Path analyses were conducted to test the hypothesized model, assuming a full mediation, that is, the hypothesis that mental health is indirectly predicted by the examined personal and environmental resources through WFF and FWF was tested, controlling for the relationships with the examined covariates. The correlations between the examined independent and mediator variables were included. The number of missing data patterns was eight. The model fit indices of the initially specified model were as follows: χ^2^ = 2520.507; df = 7; *p* = 0.000; CFI = 0.710; TLI = 0.000; RMSEA = 0.248 (90% C.I. 0.222; 0.275); and SRMR = 0.060. Only the SRMR indicated a good fit of the model to the data. The plausible modification indices suggested adding direct paths with optimism; emotional regulation—positive refocusing and planning; and enjoying free time/time for oneself. The model fit indices of this model were χ^2^ = 190.432; df = 3; *p* = 0.000; CFI = 0.981; TLI = 0.767; RMSEA = 0.098 (90% C.I. 0.059; 0.141); and SRMR = 0.009. Only the CFI and SRMR indicated a good fit. There were no plausible modification indices suggested by the program. After excluding non-significant paths (but with *p*-levels higher than 0.10), the model fit indices all suggested a good to very good fit of the model to the data as follows: χ^2^ = 290.107; df = 15; *p* = 0.016; CFI = 0.983; TLI = 0.960; RMSEA = 0.041 (90% C.I. 0.017; 0.062); and SRMR = 0.013. The resulting standardized parameter estimates (STDYX path coefficients and explained variance R2) and their significance are shown in Figure 1. The non-significant paths and the correlations are not shown due to a higher level of clarity of the display. The non-presented standardized parameter estimates (path coefficients), standard errors, and significance levels are shown in Table 4. The bootstrap simulation method was performed on this model. All the mediations considering the main variables, shown in Figure 1, are significant at the 95% significance level, which can be seen by the bootstrap confidence intervals, not including the zero value (when considering three decimal values), in Table 5.

#### Interpreting the Model Results

The results presented in Figure 1 and in Table 4 and Table 5 confirm some of the assumed relationships and mediation processes. A higher reported level of WFF is predicted by higher levels of social support at work and higher levels of education, personal strength, and recovery management (listed from the greatest to lowest contribution). The predictors explain 19% of the variance of WFF. A higher level of FWF (46% of the variance) is predicted by higher levels of social support in the family, personal strength, emotional regulation strategy of planning, and optimism; a younger age; the male gender; a greater number of children; and a higher level of education. Greater mental health (47% of the variance) is directly predicted mostly by higher levels of optimism, then by recovery management, FWF, emotional regulation strategies of positive refocusing and planning, and a greater number of children. Greater mental health is indirectly predicted by higher levels of optimism, planning, personal strength, and social support in the family through a higher level of FWF. It is vivid in Table 4 that WFF is at the sole border of significance according to the *p*-level of the path coefficient estimate in predicting mental health. However, the bootstrap 95% interval includes zero, so it could not be considered as significant. Issues of multicollinearity should be considered, especially the correlations of optimism to the other independent variable, mostly personal strength and positive refocusing, and social support in the family and at work. The correlations estimated in the model were in line with those explained previously (Table 3); however, the results of model testing show an additional significant correlation, and an older age is associated with a lower level of social support in the family (although the correlation is low; *r* = −0.085).

## 4. Discussion

The incentives for this research were the need to focus on the strengths, possibilities, and opportunities of families with a child or children with developmental disabilities [12,16], and the scarcity of quantitative studies that more comprehensively observe the positive aspects of work–family integration [6], especially in the national context [11]. Thus, the aim of this study was to examine the contributions of some individual and environmental strengths and resources (optimism; posttraumatic growth—personal strength; the cognitive emotional regulation strategies of positive reappraisal and reorienting to planning; recovery management; and social support at work and in the family) in explaining both directions of work–family facilitation and the mental health of parents of children with disabilities in Croatia. The potential mediating roles of WFF and FWF in the relationship between the examined predictors and mental health were also examined, along with the addition of some general socio-demographic covariates (gender, age, education, and number of children) to the model. This would be the first study to quantitatively examine the relationships between all the mentioned factors and the mediating roles of WFF and FWF between the resources suggested as predictors and mental health among working parents of children with developmental disabilities. The results of this study confirm some of our hypotheses about the examined relationships and mediation processes.

### 4.1. Predicting Work-to-Family Facilitation and Family-to-Work Facilitation in Parents of Children with Disabilities in Croatia

The results show that WFF is predicted by higher levels of social support at work, a higher level of education, PTG of personal strength, and recovery management, but not by optimism or the examined emotional regulation strategies, and only 19% of the variance of WFF is explained. When it comes to FWF (46%), higher levels are predicted by higher levels of social support in the family, PTG in personal strength, emotional regulation strategy of reorienting to planning, optimism, a younger age, the male gender, a greater number of children, and a higher level of education. It is not predicted by the emotional regulation strategy of positive refocusing, social support at work, or recovery management. The results are in line with the Resource–Gain–Development Perspective specifying that personal characteristics and environmental resources that promote positive domain experiences and the acquisition of gains are the key promoters of facilitation [45].

Previous research shows that domain-specific characteristics, in comparison to cross-domain characteristics, are relatively more important predictors of the effects of the same domain to another domain [67], which can explain the relative contributions of social support at work and in the family to WFF and FWF. Additionally, the measures of social support used in this study contain items referring to social support in each domain that is aimed towards issues of the other domain, so it is logical to assume that experiencing more support for family issues in the work domain would contribute to the experience that the work domain facilitates family life, and vice versa. A higher level of education could be associated with better work conditions, higher positions, greater skills, and more financial resources [68], which could be beneficial for both the work and family domains. The obtained contribution of having higher levels of being able to recover, that is, to relax and enjoy activities during one’s free time, to higher reported levels of WFF could be related to a previous finding that the coping strategy of escape that is used to deal with work stressors is positively related to WFF [69]. This could reflect that work conditions offer parents the possibilities of managing time for themselves and rewinding not only from work, but also from family obligations. This variable did not have a significant relative contribution to FWF, probably because these activities could be connected to activities with the family (so, being able to recover contributes to having more quality time with the family).

The PTG of personal strength showed to be an important resource for the experiences of both WFF and FWF. Reports of the growth of personal strength after dealing with the child’s diagnosis reflect a higher sense of self-confidence, an ability to deal with difficulties, a greater acceptance of situations, and therefore, greater possibilities to adapt and to obtain the gains and resources of both domains. Also, positive thinking and reorienting to planning on how to deal with situations contributed to the experience of the family life facilitating work life, and it is obvious that the examined personal resources were more predictive of FWF in general. One’s family and private life might depend more on personal factors since one has a higher control over what is going on in these domains. Perhaps the work domain is generally more complex to manage than the home domain, making planning behavior and staying positive while combating situations in the family relatively more important for being efficient with time and energy at work than at home [70]. The association of a greater sense of FWF to the male gender, a younger age, and more children in the family is also not surprising due to a greater vitality in younger people [71], the likely greater support of men’s work in a society that is still prone to traditional gender roles of men as the main breadwinners [72], and the results of previous studies showing greater numbers of children being related to greater work–family enrichment [51]. According to the characteristics of our sample participants, it seems that a greater number of children is related to the presence of a child/children without difficulties in the family, which has been associated with higher well-being in previous studies [73]. The presence of healthy children could be an additional source of satisfaction and support in the family, making it easier for parents to balance demands [37]. On the other hand, a lower number of children could reflect the decision of parents not to have more children due to heightened levels of care and the worry of developmental risks in another child [74].

### 4.2. Predicting Mental Health in Parents of Children with Disabilities in Croatia and the Mediational Roles of Work-to-Family Facilitation and Family-to-Work Facilitation

Higher levels of mental health (47%) were directly predicted mostly by higher levels of optimism, by recovery management, family-to-work facilitation, emotional regulation strategies of positive refocusing and planning, and a greater number of children, but not by PTG of personal strength, social support at work and in the family, or WFF. It was predicted indirectly by all the predictors of FWF, showing the significant mediating role of FWF in the relationship between these predictors and mental health; higher levels of optimism, the ER strategy of planning, PTG of personal strength, and social support in the family predicted greater mental health through a higher level of FWF. Neither the predictive nor mediating role of WFF in explaining mental health and its relationships with the examined personal and environmental resources were confirmed, although the contribution is very near to significance.

The protective role of optimism in different aspects of the mental health of parents of children with disabilities was documented earlier [37,39]. It could be said that this personality trait helps parents to better deal with negative experiences, and helps with growth after such experiences along with adopting health-promoting behaviors and adaptable coping strategies [75]. They also reflect their hope and positivity to others, and thus have better social relationships, which are all good for mental health [76]. Related to this are the results showing the beneficial contribution of the examined cognitive emotional regulation strategies as adaptive coping strategies that reflect one’s orientation to the positive aspects of situations and ways to manage them. Recovery management in parents of children with a developmental disability is viewed as a health-promoting behavior as well [77], and reflects a manner of self-care [78,79]. A meta-analysis (k  =  316; independent samples; *N*  =  99,329 participants) showed that relaxation and mastery experiences positively predict personal outcomes, such as positive effects, life satisfaction, and well-being [32], which are both comprehended in the measure of recovery management in this study.

The relatively greater importance of family-to-work facilitation for mental health was shown in previous studies, and was shown to be a family protective factor that buffers the detrimental effects of work–family conflict on mental health [44]. Through the provision of support and love, families can act as a bridge between their working members and the organization/industry in facilitating mental health care [80]. The number of children as a significant predictor of mental health could be explained with the same suggested mechanisms as the ones for predicting FWF (greater support in the family, a reflection of less stress in the family to have more children, and a sense of hope). The mentioned factors shown to predict WFF, FWF, and mental health in this study are also described through the Family Resilience Theory [81,82], defining family resilience as the capacity of the family to maintain a level of healthy functioning during crisis situations. The theory distinguishes protective family characteristics (employment, the distribution of duties within the family, the financial status of the family, joint agreements, and cohesion), protective processes (spousal support, communication, problem solving, and warm supportive relationships), and recovery factors (support in crisis situations, optimism, and environmental support). Work–family policies and educative (informative) or training programs that encourage the studied personal and environmental resources should be designed and applied to promote the work–life balance and mental health of employed parents of children with a developmental disability, which would benefit their families, organizations, and the wider community.

### 4.3. Limitations and Future Research

Aside from the advantages of this study that were pointed out throughout the paper, some shortcomings should be pointed out. Although this study included a relatively large number of employed parents of children with disabilities, participation was voluntary and did not include compensation. Therefore, there is a possibility that those with a greater lack of time, energy, and resources; greater issues of work–family balance and different life role imbalances; and heightened stress levels were underrepresented in the study, that is, they could not manage to fill out the questionnaire. On the other hand, the participants may have been very motivated to share their problems related to raising children with disabilities, also limiting the generalizability of the results. Male participants were underrepresented in the study, and there were several participants with children without an official diagnosis. In addition, we did not control whether the male and female participants were partners, that is, parents of the same child/ren. Future research should be attempted on more representative samples of the parents in question, living in Croatia, using population sampling techniques. Dyadic relationships could be considered as well, for example, the work–family fit of the spouse/partner.

The self-assessment nature of data collection also makes the objectivity and reliability of the data difficult to obtain. Assessments from other families and/or organizational members could be collected, or intervention and observational or diary studies could be conducted. Also, the research was cross-sectional with data collected in one time point; thus, the data analysis was correlational, making it impossible to give conclusions on any causal relationships. The relationships between the examined variables could be reciprocal or in the opposite direction; for instance, better mental health could be a factor influencing the perceptions of work–family facilitation instead [83]. Also, hypotheses on more specific relationships between the independent variables (besides only correlational relationships) could be tested, for example, whether optimism contributes to posttraumatic growth and more adaptive coping strategies, or whether it is the other way around [75]. Moderating relationships among the same variables and covariates could also be considered.

Other possible and more independent (to avoid issues of multicollinearity shown in this study), personal, and environmental resources could also be observed, especially in the workplace, considering the low explained variance of WFF. Future research could consider other personal characteristics such as other potentially strengthening or adaptive personality traits, other cognitive or behavioral coping strategies, more specific activities during “time for self”, preferred ways of managing work and family boundaries, work characteristics such as control and flexibility at work, specific family-friendly organizational policies, positive work culture, work efficacy, etc.

## 5. Conclusions

In this study, we decided to focus on the positive aspects and explore some personal and environmental strengths and resources that could promote the experiences of work–family facilitation and the mental health of parents of children with disabilities in the Croatian national context. The path analysis showed that, among the main variables of the study, a higher level of mental health (47% of the variance explained) was directly predicted mostly by higher levels of optimism, recovery management, family-to-work facilitation (FWF), emotional regulation strategies of positive refocusing and planning, and a greater number of children. It was also predicted indirectly by all the predictors of FWF (46%) (higher levels of optimism, ER strategy of planning, PTG of personal strength, and social support in the family) through a higher level of FWF. It was not significantly predicted by work-to-family facilitation (WFF), which was predicted by PTG of personal strength and recovery management (19% of the variance). The tested model included the covariates of gender, age, education, and number of children, showing some significant but relatively low contributions. Work–family policies and educative (informative) or training programs that encourage the studied personal and environmental resources should be designed and applied to promote the work–life balance and mental health of employed parents of children with a developmental disability, which would benefit their families, organizations, and the wider community. Further research should be conducted to advance knowledge about the positive aspects and promoters of the quality of life of parents with a child who needs special care.

## Figures and Tables

**Table 1:** Description of the sample participants (*N* = 571).

Characteristics	Descriptive Parameters
Age	*M* = 40.9, *SD* = 6.15, Min = 23, Max = 66
Education	Elementary school (*n* = 4); Secondary school (*n* = 237); Bachelor’s degree (*n* = 90) Master’s degree (*n* = 205) Doctoral degree (*n* = 35)
Marital status	Extramarital union (*n* = 57) Married (*n* = 450) Separated or divorced (*n* = 46) Single (*n* = 14) Widow/er (*n* = 4)
Number of children	Mode = 2 (f = 267), Min = 1, Max = 6
Number of children with disabilities	Mode = 1 (f = 514); Min = 1, Max = 4
Highest degree of disability among children	4th— most severe level of impairment (*n* = 204) 3rd—severe level of impairment (*n* = 158) 2nd—moderate level of impairment (*n* = 70) 1st—mild level of impairment (*n* = 35) Do not know/not sure (*n* = 104)
Employment sector	Public (*n* = 265) Private (*n* = 306)
Partner’s employment	Yes (*n* = 463) No (*n* = 24) Caregiver (*n* = 20)

**Table 2:** Descriptive parameters of the analyzed variables (*N*_total_ = 571).

	*N*	*M*	*SD*	Min	Max	Skewness	Kurtosis
Gender (0—men; 1—women)	571	0.91	0.29	0.00	1.00	−2.78	5.74
Age	564	40.90	6.15	23.00	66.00	0.27	0.24
Education	571	3.05	1.02	1.00	5.00	0.20	−1.35
Number of children	571	2.20	0.99	1.00	6.00	1.05	1.57
Optimism	571	3.62	0.70	1.00	5.00	−0.76	1.09
Emotional regulation—positive refocusing	571	3.24	0.86	1.00	5.00	−0.26	−0.09
Emotional regulation—planning	571	4.05	0.64	1.00	5.00	−0.62	1.34
Posttraumatic growth—personal strength	571	3.31	1.09	0.00	5.00	−0.92	0.88
Recovery management	568	2.80	1.06	1.00	5.00	0.15	−0.68
Social support at work	565	4.98	1.26	1.00	7.00	−0.72	0.61
Social support in the family	566	5.27	1.27	1.25	7.00	−0.66	−0.15
Work-to-family facilitation	566	2.91	0.83	1.00	5.00	−0.06	−0.09
Family-to-work facilitation	563	3.41	0.82	1.00	5.00	−0.27	−0.03
Mental health	571	3.75	0.86	1.00	6.00	−0.49	0.09

**Table 3:** Pearson’s coefficients of correlations between the analyzed variables (*N* = 555; case-wise deletion of missing data).

	2	3	4	5	6	7	8	9	10	11	12	13	14
1. Gender (0—men; 1—women)	**−0.10**	−0.01	−0.04	0.04	0.04	0.06	0.05	−0.15	−0.00	−0.08	0.00	**−0.09**	**−0.09**
2. Age	-	**0.10**	**0.12**	−0.07	−0.04	−0.05	−0.05	**0.09**	−0.07	−0.08	0.01	**−0.17**	−0.04
3. Education		-	**−0.15**	−0.04	**−0.10**	−0.01	**−0.10**	−0.05	0.03	−0.00	**0.15**	0.00	−0.05
4. Number of children			-	**0.16**	**0.12**	**0.09**	**0.13**	0.04	0.05	0.06	−0.02	**0.13**	**0.17**
5. Optimism				-	**0.47**	**0.34**	**0.52**	**0.35**	**0.36**	**0.39**	**0.27**	**0.45**	**0.58**
6. ER—positive refocusing					-	**0.19**	**0.38**	**0.23**	**0.16**	**0.20**	**0.17**	**0.26**	**0.40**
7. ER— planning						-	**0.26**	0.05	**0.13**	**0.15**	**0.11**	**0.29**	**0.11**
8. PTG—personal strength							-	**0.26**	**0.22**	**0.25**	**0.25**	**0.42**	**0.44**
9. Enjoying time for oneself								-	**0.32**	**0.38**	**0.26**	**0.29**	**0.46**
10. Social support at work									-	**0.48**	**0.33**	**0.35**	**0.29**
11. Social support in the family										-	**0.18**	**0.56**	**0.42**
12. Work-to-family facilitation											-	**0.35**	**0.18**
13. Family-to-work facilitation												-	**0.42**
14. Mental health													-

Note: Correlations in bold are significant at the *p* < 0.05 level. ER—emotional regulation; PTG—posttraumatic growth.

**Table 4:** Standardized (STDYX) parameter estimates, standard errors, and significance levels for the examined model paths and correlations not shown in Figure 1 (*N* = 571).

Model Relationship	Standardized Estimate (STDYX)	Standard Error (STDYX)	Two-Tailed *p*-Value (STDYX)
Non-significant path coefficients			
Optimism → Work-to-family facilitation	0.090	0.050	0.084
Work-to-family facilitation → Mental health	−0.073	0.037	0.051

**Table 5:** Non-standardized estimates of the indirect effects.

Indirect Effects:	Non-Standardized Estimates of the Indirect Effect	95% Confidence Interval (k = 1000)
Optimism → Family-to-work facilitation → Mental health	0.020	0.005; 0.046
Emotional regulation: Planning → Family-to-work facilitation → Mental health	0.042	0.019; 0.074
Posttraumatic growth: Personal strength → Family-to-work facilitation → Mental health	0.041	0.021; 0.069
Social support in the family → Family-to-work facilitation → Mental health	0.070	0.040; 0.105

Note. k—number of bootstrapped samples.

## Data Availability

The data are available upon request from the research authors.
